# Statistical Genomic Approach Identifies Association between FSHR Polymorphisms and Polycystic Ovary Morphology in Women with Polycystic Ovary Syndrome

**DOI:** 10.1155/2015/483726

**Published:** 2015-07-26

**Authors:** Tao Du, Yu Duan, Kaiwen Li, Xiaomiao Zhao, Renmin Ni, Yu Li, Dongzi Yang

**Affiliations:** ^1^Department of Gynecology & Obstetrics, Sun Yat-sen Memorial Hospital, Sun Yat-sen University, 107 West Yanjiang Road, Guangzhou 510120, China; ^2^Department of Urology, Sun Yat-sen Memorial Hospital, Sun Yat-sen University, Guangzhou, China

## Abstract

*Background.* Single-nucleotide polymorphisms (SNPs) in the follicle stimulating hormone receptor (FSHR) gene are associated with PCOS. However, their relationship to the polycystic ovary (PCO) morphology remains unknown. This study aimed to investigate whether PCOS related SNPs in the FSHR gene are associated with PCO in women with PCOS. *Methods*. Patients were grouped into PCO (*n* = 384) and non-PCO (*n* = 63) groups. Genomic genotypes were profiled using Affymetrix human genome SNP chip 6. Two polymorphisms (rs2268361 and rs2349415) of FSHR were analyzed using a statistical approach. *Results*. Significant differences were found in the allele distributions of the GG genotype of rs2268361 between the PCO and non-PCO groups (27.6% GG, 53.4% GA, and 19.0% AA versus 33.3% GG, 36.5% GA, and 30.2% AA), while no significant differences were found in the allele distributions of the GG genotype of rs2349415. When rs2268361 was considered, there were statistically significant differences of serum follicle stimulating hormone, estradiol, and sex hormone binding globulin between genotypes in the PCO group. In case of the rs2349415 SNP, only serum sex hormone binding globulin was statistically different between genotypes in the PCO group. *Conclusions*. Functional variants in FSHR gene may contribute to PCO susceptibility in women with PCOS.

## 1. Introduction

Polycystic ovary syndrome (PCOS) affects about 4%–12% women within reproductive age [1]. It is characterized by hyperandrogenism, menstrual irregularity, infertility, and polycystic ovarian (PCO) morphology [2, 3]. Although the term PCO is used, not all patients have polycystic ovaries [4, 5]. Ultrasonography is the only way to detect PCO, the results of which may be related to the experience of doctors, the definition of devices, and the detected time of the menstruation period.

The pathogenesis of PCO and PCOS is still not completely understood. Recent studies report that PCOS may be associated with single-nucleotide polymorphisms (SNP). Our previous GWAS identified two SNPs (rs2268361 and rs2349415) in the follicle stimulating hormone receptor (FSHR) gene associated with PCOS in the Han Chinese population [6]. FSHR is specifically expressed by the granulosa cells of the ovary and bound by its ligand serum follicle stimulating hormone (FSH), which mediates the functions of FSH and plays important roles in the folliculogenesis by affecting the maturation and development of follicles [7, 8]. If FSHR is null, females would be sterile, with small ovaries and blocked follicular development [9].

While rs2268361 and rs2349415 SNPs are confirmed to be associated with PCOS, their relationship to the PCO morphology remains unknown. The present study performs a case-control study in the Han Chinese women with PCOS in South China and investigates the association between the two FSHR gene SNPs and the PCO morphology.

## 2. Methods

### 2.1. Subjects

Patients with PCOS were recruited by Reproductive Medicine Center, Sun Yat-sen Memorial Hospital of Sun Yat-sen University from January 2010 to August 2011. All subjects were unrelated Han Chinese from south China. The study was approved by the local Medical Ethical Committee and all participants have written consented for the purpose of the study.

The patients were selected based on the 2003 Rotterdam Diagnostic Criteria [2], requiring the presence of any two of the following three criteria: oligoovulation and/or anovulation; clinical and/or biochemical signs of hyperandrogenism; and polycystic ovary morphology (PCO) and exclusion of other etiologies. Patients were excluded if they: (a) used hormonal medication, including oral contraceptives, for at least 3 months prior to starting the study; (b) had other endocrine disorders or known causes of hyperandrogenism, such as thyroid abnormalities, congenital adrenal hyperplasia, Cushing syndrome, and androgen-secreting tumors; (c) had previous ovary-related surgery, chemotherapy, or radiotherapy; and (d) had pregnancy. Patients were classified into PCO or non-PCO groups according to the ovary appearance on ultrasound. A finding of PCO was determined when 12 or more follicles measuring 2–9 mm in diameter were scanned in either ovary or the ovarian volume was greater than 10 mL [2].

### 2.2. Clinical Measurements

Detailed medical information of included patients was collected, including the parameters age, body mass index (BMI), waist-hip ratio (WHR), transvaginal ultrasound results, modified Ferriman-Gallwey score (mF-G score) [10], and acne score [11]. Transvaginal ultrasonography was performed on cycle days 3–5 (or between days 3 and 5 after a progestin-induced withdrawal bleeding for the amenorrhea patients).

### 2.3. Biochemical Measurements

On cycle days 3–5 (or between days 3 and 5 after a progestin-induced withdrawal bleeding for the amenorrhea patients), venous blood samples were taken between 8 a.m. and 9 a.m. after a 12 h overnight fast. The serum hormone levels, including follicle stimulating hormone (FSH), luteinizing hormone (LH), prolactin (PRL), estradiol (E2), total testosterone (TT), androstenedione (A2), free testosterone (FT), dehydroepiandrosterone sulfate (DHEA-S), and estrone (E1), were measured by the chemiluminescence immunoassays Access 2 (Beckman, Fullerton, CA). Sex hormone binding globulin (SHBG) and 17-hydroxyprogesterone (17-OHP) were measured using the Access 2 ELISA kit (Beckman, Fullerton, CA). Serum cholesterol (CHOL), triglycerides (TG), high-density lipoprotein-cholesterol (HDL-C), and low-density lipoprotein (LDL-C) levels were measured using an enzymatic calorimetric method (Hitachi 7600). Plasma glucose was detected by the glucose oxidase method (Hitachi 7600), and insulin was detected by chemiluminescence immunometric assay and commercial kit (Immulite2000 Analyzer; CPC). The homeostatic model assessment for insulin resistance (HOMA-IR), insulin sensitivity (HOMA-IS), and *β* (HOMA-*β*) was calculated as follows: (fasting insulin [mIU/L] × fasting glucose [mmol/L])/22.5, 1/(fasting insulin [mIU/L] × fasting glucose [mmol/L]), 20 × fasting insulin [mIU/L]/(fasting glucose [mmol/L] − 3.5) (%), respectively.

### 2.4. Genotyping

The detailed procedures of genotyping were referred to in the GWAS study [6]. EDTA anticoagulated venous blood samples were collected from all patients. Genomic DNA was extracted from peripheral blood lymphocytes by using Flexi Gene DNA kits (Qiagen, Germany) and was diluted to working concentrations of 50 ng/*μ*L for genome-wide genotyping and 15–20 ng/*μ*L for the validation study. Genotyping was performed using Affymetrix human genome SNP chip 6. The two polymorphisms (rs2268361 and rs2349415) of FSHR were selected according to the previous criteria [6, 12].

### 2.5. Statistical Analysis

Statistical analysis was performed using Stata/SE 12.0 (StataCorp LP, TX, USA). Continuous variables were compared using Student's *t*-test or ANOVA for parametric data and Mann-Whitney test or Kruskal-Wallis test for nonparametric data. Categorical data were compared using Chi-squared test. Genotype frequencies were tested for Hardy-Weinberg equilibrium. Linkage disequilibrium (LD) was estimated with *D*′ and *r* ^2^ on SHEsis (http://analysis.bio-x.cn/myAnalysis.php). All tests were considered statistically significant at *P* < 0.05.

## 3. Results

A total of 447 women with PCOS met the eligible criteria for the study. The mean age was 27.13 ± 3.88. Among them, 384 women had PCO, but 63 did not. The baseline characteristics of the subjects were shown in Table 1. There was no statistically significant difference in age, BMI, WHR, acne score, mF-G score, HOMA-IR, HOMA-ISI, HOMA-*β*%, serum CHOL, TG, HDL-C, FSH, LH, PRL, E2, TT, A2, DHEA-S, E1, and 17-OHP levels between the PCO and non-PCO groups. However, serum LDL-C and FT were significantly higher in PCO group compared with non-PCO group, while serum SHBG level was significantly lower (*P* = 0.02, 0.04, and 0.04, resp.) (Table 1).

The allele and genotype frequencies for rs2268361 and rs2349415 SNPs in PCOS women with or without PCO were shown in Table 2. The genotype frequencies of the two SNPs were in accordance with the Hardy-Weinberg equilibrium (*P* = 0.5763 and 0.6452, resp.). PCOS women with genotype G/A had significantly higher risk of suffering from PCO (*P* = 0.03). Therefore, polymorphisms of rs2268361 were associated with PCO morphology. However, there were no statistical difference in the allele frequencies of rs2268361 and in the allele or genotype frequencies of rs2349415 between PCO group and non-PCO group. The extent of linkage disequilibrium in pairwise combinations of the two SNPs was calculated. Among all the PCOS women, linkage disequilibrium (*D*′) between the rs2268361 and rs2349415 SNPs of FSHR gene was 0.610 (*r* ^2^ = 0.113), indicating a low linkage of the two polymorphisms.

Further analyses of phenotypes were performed for the rs2268361 and rs2349415 polymorphisms in both the PCO group and non-PCO group (Tables 3 and 4). When the rs2268361 SNP was considered, there were statistically significant differences in the levels of serum FSH (*P* = 0.02), E2 (*P* = 0.03), and SHBG (*P* = 0.03) between genotypes in the PCO women. However, no serum hormones were significantly different between genotypes in the non-PCO group. In case of the rs2349415 SNP, only the level of serum SHBG (*P* = 0.02) was statistically different between genotypes in the PCO group.

## 4. Discussion

Polycystic ovary syndrome is a complex hereditary endocrine disorder with complicated clinical manifestations. Higher FT and lower SHBG in serum were detected in PCO group compared with non-PCO group, indicating that more actions of androgen might be associated with folliculogenesis. Polymorphisms (rs2268361, but not rs2349415) in FSHR gene were found to be associated with the presentation of PCO.

FSHR, with its ligand FSH, is one of the main factors affecting PCOS. It is reported that FSHR is highly expressed in granulosa and ovarian surface epithelium cells from stimulated follicles of women with PCOS [13, 14]. It is a member of G protein-coupled receptors family including three domains, which are extracellular ligand-binding, transmembrane, and intracellular domains [12, 15]. FSHR gene is located on chromosome 2p21 and comprises 10 exons and 9 introns [16]. Overexpression of FSHR may lead to enhanced proliferation in ovarian surface epithelial cells, therefore generating more premature follicles [17]. In addition, the level of FSH is also controlled by FSHR and aberrant FSHR may affect ovarian folliculogenesis.

As several PCOS susceptibility genes including INS VNTR, TCF7L2, and PPARG [18–20], polymorphisms or SNPs for the FSHR gene may also be associated with PCOS. Many studies have focused on investigating the most controversial SNPs in FSHR gene, which are Thr307Ala and Asn680Ser polymorphisms in exon 10 [21]. The latest systematic review reveals that there exist no associations between the two SNPs and PCOS [21]. However, the sample size of this meta-analysis is relatively small, which may not have abundant statistical power in assessing the role of FSHR polymorphisms in the development of PCOS [21]. Our previous study analyzed genome-wide association data from two cohorts of Han Chinese in large sizes with 744 PCOS cases and 895 controls in GWAS 1 and 1,510 PCOS cases and 2,016 controls in GWAS 2. The study also followed up significantly associated signals identified in the combined results of GWAS 1 and 2 in a total of 8,226 cases and 7,578 controls. Among the top ten significant signals, two SNP signals (rs2268361 and rs2349415) located in the FSHR gene were identified to be associated with PCOS [6].

Women with PCOS can be diagnosed without PCO morphology according to the 2003 Rotterdam Criteria [2]. PCO appearance under ultrasound is only detected in 62.7% to 91% PCOS women [5, 22]. There are essentially two anomalies affecting folliculogenesis [23]: excessive small follicular growth and the defective selection of a dominant follicle. Androgens are primarily responsible for promoting the growth of small follicles [24, 25], and adequate action of FSH is essential for selecting a dominant follicle. When the secretion or function of FSH is inhibited, PCO will be presented [26]. However, it is a common belief that women with PCOS have serum FSH levels at the same range as normal women in early follicular phase [27]. Therefore, the function of FSH via combining to FSHR may be the most important. In the present study, significantly higher FT and lower SHBG in PCO than non-PCO patients might initially promote the growth of small follicles. One of two analyzed SNP (rs2268361) in the FSHR gene was significantly associated with PCO. Patients with G/A at rs2268361 had high risk of presenting PCO. SNP at rs2268361 in the FSHR gene might result in changing the reaction to FSH stimulation, while FSH was similar between PCO and non-PCO groups. The function of FSHR in PCO-group women with G/A at rs2268361 might be defected comparing with the other two patterns, while serum estradiol was also significantly lower in G/A pattern. No association between SNP (rs2349415) in the FSHR gene and PCO morphology was detected.

The main limitation of this study is the relative small sample size. Further studies in larger populations are needed to come up with more supporting data. Furthermore, because of differences in ethnicity and sample size, effects of the SNPs in the FSHR genes could account for some conflicting results. However, to our knowledge, this study is the first to identify the association between SNPs in the FSHR gene and PCO morphology in PCOS women. It can bring a new insight into the cause and diagnosis of PCO.

## 5. Conclusions

This is the first study to identify the association between SNP (rs2268361) in the FSHR gene and PCO morphology in PCOS women. PCOS women with G/A at rs2268361 SNP will have higher risk of appearing PCO. There is no association between SNP (rs2349415) in the FSHR gene and PCO appearance. Further studies with populations in other ethnicities and larger size are needed to help clarify the PCO susceptible SNPs in the FSHR gene.

## Figures and Tables

**Table 1:** Demographic characteristics and endocrine profiles of PCOS women with or without polycystic ovary morphology.

	Non-PCO	PCO	*P* value^*^
Case number	63	384	—
Age, year	26.86 ± 3.65	27.18 ± 3.92	0.55
BMI, kg/m^2^	23.28 ± 9.47	22.83 ± 4.70	0.74
WHR	0.84 ± 0.21	0.82 ± 0.07	0.67
Acne score	1 (0–4)	0 (0–5)	**0.049**
mF-G score	6 (0–22)	4 (0–35)	0.35
CHOL, mmol/L	4.73 ± 0.75	4.97 ± 0.87	0.08
TG, mmol/L	1.26 ± 0.73	1.32 ± 0.84	0.67
HDL-C, mmol/L	1.62 ± 0.48	1.57 ± 0.44	0.51
LDL-C, mmol/L	2.62 ± 0.67	2.92 ± 0.82	**0.02**
HOMA-IR	2.19 ± 1.72	2.34 ± 1.98	0.59
HOMA-ISI	0.03 ± 0.02	0.03 ± 0.03	0.71
HOMA-*β*%	135.96 ± 115.58	131.87 ± 130.32	0.82
FSH, IU/L	6.68 ± 2.34	6.46 ± 2.33	0.51
LH, IU/L	10.24 ± 7.80	10.88 ± 6.99	0.53
PRL, ng/mL	16.00 ± 10.47	16.63 ± 11.20	0.70
E2, ng/L	84.62 ± 93.91	71.45 ± 75.47	0.34
TT, nmol/L	2.41 ± 1.02	2.40 ± 1.31	0.99
A2, ng/mL	4.80 ± 2.22	5.22 ± 2.46	0.37
FT, pg/mL	3.65 ± 3.01	4.67 ± 5.68	**0.04**
DHEA-S, ng/mL	2245.25 ± 1009.03	2159.46 ± 1007.76	0.54
E1, pg/mL	101.77 ± 37.95	117.18 ± 83.34	0.23
SHBG, nmol/L	85.10 ± 72.80	64.20 ± 55.802	**0.04**
17-OHP, ng/mL	1.17 ± 1.00	1.27 ± 0.87	0.43

^*^Significant difference was in bold.

PCO: polycystic ovary; BMI: body mass index; WHR: waist-hip ratio; mF-G score: modified Ferriman-Gallwey score; CHOL: cholesterol; TG: triglycerides; HDL-C: high-density lipoprotein-cholesterol; LDL-C: low-density lipoprotein-cholesterol; HOMA-IR/HOMA-ISI/HOMA-*β*%: homeostatic model assessment for insulin resistance/insulin sensitivity/*β*; FSH: follicle stimulating hormone; LH: luteinizing hormone; PRL: prolactin; E2: estradiol; TT: total testosterone; A2: androstenedione; FT: free testosterone; DHEA-S: dehydroepiandrosterone sulfate; E1: estrone; SHBG: sex hormone binding globulin; 17-OHP: 17-hydroxyprogesterone.

**Table 2:** Genotype distribution and allele frequency of FSHR polymorphisms in PCO women with or without polycystic ovary.

rs2268361			*P* value^*^	H-W test	rs2349415			*P* value	H-W test
	PCO	Non-PCO				PCO	Non-PCO		
GG	106	21	**0.03**	0.5763	CC	202	39	0.38	0.6452
GA	205	23			CT	156	21		
AA	73	19			TT	26	3		
G	417	65	0.63		C	560	99	0.18	
A	351	61			T	208	27		

^*^Significant difference was in bold.

PCO: polycystic ovary; H-W test: Hardy-Weinberg equilibrium test.

**Table 3:** FSHR polymorphisms versus endocrine parameters in PCO patients.

		rs2268361		*P* value^*^		rs2349415		*P* value^*^
	GG	GA	AA		CC	CT	TT	
Case number	106	205	73	—	202	156	26	—
Age, year	26.82 ± 3.95	27.45 ± 3.67	26.92 ± 4.52	0.33	27.19 ± 4. 16	26.96 ± 3.70	28.42 ± 3.15	0.21
BMI, kg/m^2^	22.67 ± 4.75	22.90 ± 4.91	22.89 ± 3.98	0.92	22.81 ± 4.32	22.83 ± 5.12	23.00 ± 5.02	0.98
WHR	0.82 ± 0.08	0.83 ± 0.08	0.82 ± 0.07	0.82	0.82 ± 0.08	0.82 ± 0.07	0.82 ± 0.06	0.99
Acne score	1 (0–4)	0 (0–5)	0 (0–5)	0.07	0 (0–4)	0 (0–5)	1 (0–4)	0.63
mF-G score	5 (0–27)	4 (0–35)	3.5 (0–22)	0.12	4 (0–35)	4 (0–29)	5.5 (0–16)	0.33
CHOL, mmol/L	4.89 ± 0.82	4.99 ± 0.88	5.04 ± 0.90	0.54	4.93 ± 0.89	5.01 ± 0.88	5.03 ± 0.69	0.69
TG, mmol/L	1.30 ± 0.82	1.37 ± 0.91	1.22 ± 0.62	0.44	1.27 ± 0.89	1.27 ± 0.89	1.39 ± 0.76	0.45
HDL-C, mmol/L	1.60 ± 0.42	1.58 ± 0.46	1.51 ± 0.42	0.42	1.53 ± 0.40	1.62 ± 0.49	1.56 ± 0.41	0.21
LDL-C, mmol/L	2.81 ± 0.76	2.93 ± 0.84	3.04 ± 0.84	0.21	2.94 ± 0.84	2.86 ± 0.83	3.02 ± 0.59	0.58
HOMA-IR	2.09 ± 1.78	2.45 ± 2.10	2.39 ± 1.91	0.30	2.29 ± 1.75	2.44 ± 2.32	2.12 ± 1.38	0.66
HOMA-ISI	0.03 ± 0.02	0.03 ± 0.03	0.03 ± 0.02	0.82	0.03 ± 0.03	0.03 ± 0.02	0.03 ± 0.03	0.97
HOMA-*β*%	120.98 ± 156.82	135.70 ± 121.63	136.74 ± 111.78	0.61	125.02 ± 125.61	140.06 ± 137.64	135.56 ± 122.83	0.56
FSH, IU/L	6.58 ± 2.16	6.65 ± 2.50	5.72 ± 1.90	**0.02**	6.31 ± 2.12	6.58 ± 2.65	6.82 ± 1.63	0.41
LH, IU/L	11.65 ± 7.47	10.62 ± 6.73	10.42 ± 7.02	0.41	10.40 ± 6.62	11.26 ± 7.17	12.11 ± 8.48	0.36
PRL, ng/mL	16.05 ± 10.02	17.46 ± 12.46	15.04 ± 8.60	0.28	16.11 ± 10.44	16.88 ± 11.78	19.19 ± 13.29	0.45
E2, ng/L	72.88 ± 67.09	63.21 ± 63.91	92.84 ± 106.84	**0.03**	71.16 ± 76.83	71.96 ± 64.84	70.74 ± 115.86	0.99
TT, nmol/L	2.43 ± 1.24	2.41 ± 1.46	2.35 ± 0.94	0.92	2.36 ± 1.07	2.50 ± 1.61	2.09 ± 0.98	0.29
A2, ng/mL	5.03 ± 2.20	5.37 ± 2.62	5.07 ± 2.42	0.71	5.18 ± 2.29	5.43 ± 2.80	4.34 ± 1.50	0.39
FT, pg/mL	4.36 ± 5.62	5.04 ± 6.34	4.11 ± 3.49	0.41	4.76 ± 5.81	4.78 ± 5.81	2.92 ± 2.04	0.37
DHEA-S, ng/mL	2124.35 ± 1049.61	2178.40 ± 989.35	2158.74 ± 1009.01	0.91	2165.36 ± 924.47	2206.65 ± 1103.63	1774.35 ± 1013.97	0.18
E1, pg/mL	124.49 ± 71.96	126.03 ± 98.92	87.97 ± 57.90	0.29	119.74 ± 73.46	116.73 ± 102.42	105.78 ± 72.28	0.91
SHBG, nmol/L	76.09 ± 72.20	61.64 ± 51.50	53.97 ± 33.05	**0.03**	56.46 ± 35.97	73.13 ± 73.71	71.07 ± 48.71	**0.02**
17-OHP, ng/mL	1.25 ± 1.04	1.26 ± 0.81	1.33 ± 0.76	0.82	1.33 ± 0.99	1.22 ± 0.74	1.13 ± 0.50	0.44

^*^Significant difference was in bold.

PCO: polycystic ovary; BMI: body mass index; WHR: waist-hip ratio; mF-G score: modified Ferriman-Gallwey score; CHOL: cholesterol; TG: triglycerides; HDL-C: high-density lipoprotein-cholesterol; LDL-C: low-density lipoprotein-cholesterol; HOMA-IR/HOMA-ISI/HOMA-*β*%: homeostatic model assessment for insulin resistance/insulin sensitivity/*β*; FSH: follicle stimulating hormone; LH: luteinizing hormone; PRL: prolactin; E2: estradiol; TT: total testosterone; A2: androstenedione; FT: free testosterone; DHEA-S: dehydroepiandrosterone sulfate; E1: estrone; SHBG: sex hormone binding globulin; 17-OHP: 17-hydroxyprogesterone.

**Table 4:** FSHR polymorphisms versus endocrine parameters in non-PCO patients^*^.

	rs2268361			*P* value
	GG	GA	AA	
Case number	21	23	19	—
Age, year	27.05 ± 3.57	26.69 ± 4.76	26.84 ± 2.00	0.95
BMI, kg/m^2^	21.98 ± 4.13	24.89 ± 14.51	23.73 ± 4.93	0.64
WHR	0.81 ± 0.05	0.88 ± 0.34	0.81 ± 0.07	0.56
Acne score	1 (0–3)	1 (0–3)	1 (0–4)	0.63
mF-G score	7 (0–22)	5 (0–21)	4 (0–16)	0.55
CHOL, mmol/L	4.52 ± 0.77	4.68 ± 0.62	5.05 ± 0.85	0.18
TG, mmol/L	1.42 ± 1.09	1.22 ± 0.47	1.10 ± 0.35	0.50
HDL-C, mmol/L	1.76 ± 0.73	1.54 ± 0.24	1.54 ± 0.30	0.34
LDL-C, mmol/L	2.43 ± 0.77	2.54 ± 0.53	3.00 ± 0.63	0.06
HOMA-IR	2.66 ± 1.92	1.96 ± 1.94	1.96 ± 0.94	0.36
HOMA-ISI	0.03 ± 0.02	0.04 ± 0.03	0.03 ± 0.03	0.40
HOMA-*β*%	172.07 ± 121.06	120.98 ± 134.90	113.68 ± 65.57	0.25
FSH, IU/L	6.75 ± 1.91	7.25 ± 2.66	5.82 ± 2.17	0.18
LH, IU/L	13.73 ± 10.21	8.87 ± 6.39	8.29 ± 5.21	0.07
PRL, ng/mL	12.61 ± 5.76	19.79 ± 12.53	13.64 ± 9.45	0.06
E2, ng/L	83.42 ± 77.05	85.15 ± 74.46	84.97 ± 132.92	1.00
TT, nmol/L	2.14 ± 1.07	2.75 ± 0.73	2.26 ± 1.19	0.11
A2, ng/mL	4.08 ± 2.16	5.44 ± 2.39	4.56 ± 1.89	0.33
FT, pg/mL	3.32 ± 2.79	3.85 ± 3.22	3.80 ± 3.14	0.82
DHEA-S, ng/mL	2207.05 ± 1064.78	2523.01 ± 1031.51	1916.65 ± 842.80	0.17
E1, pg/mL	100.18 ± 45.10	101.58 ± 40.50	103.79 ± 31.81	0.99
SHBG, nmol/L	89.91 ± 81.37	83.32 ± 75.70	80.99 ± 58.88	0.93
17-OHP, ng/mL	1.23 ± 1.27	1.26 ± 1.00	0.94 ± 0.41	0.65

^*^No analysis was performed for rs2349415 SNP because the size of genotype pattern TT was too small.

PCO: polycystic ovary; BMI: body mass index; WHR: waist-hip ratio; mF-G score: modified Ferriman-Gallwey score; CHOL: cholesterol; TG: triglycerides; HDL-C: high-density lipoprotein-cholesterol; LDL-C: low-density lipoprotein-cholesterol; HOMA-IR/HOMA-ISI/HOMA-*β*%: homeostatic model assessment for insulin resistance/insulin sensitivity/*β*; FSH: follicle stimulating hormone; LH: luteinizing hormone; PRL: prolactin; E2: estradiol; TT: total testosterone; A2: androstenedione; FT: free testosterone; DHEA-S: dehydroepiandrosterone sulfate; E1: estrone; SHBG: sex hormone binding globulin; 17-OHP: 17-hydroxyprogesterone.

## Abbreviations

A2: Androstenedione
BMI: Body mass index
CHOL: Cholesterol
DHEA-S: Dehydroepiandrosterone sulfate
E1: Estrone
E2: Estradiol
FSH: Follicle stimulating hormone
FSHR: Follicle stimulating hormone receptor
FT: Free testosterone
HDL-C: High-density lipoprotein-cholesterol
HOMA-IR/HOMA-ISI/HOMA-*β*%: Homeostatic model assessment for insulin resistance/insulin sensitivity/*β*
LDL-C: Low-density lipoprotein-cholesterol
LH: Luteinizing hormone
mF-G score: Modified Ferriman-Gallwey score
PCO: Polycystic ovary
PCOS: Polycystic ovary syndrome
PRL: Prolactin
SHBG: Sex hormone binding globulin
SNP: Single nucleotide polymorphism
TG: Triglycerides
TT: Total testosterone
WHR: Waist-hip ratio
17-OHP: 17-Hydroxyprogesterone.
